# Natural and Synthetic Polymeric Biomaterials for Application in Wound Management

**DOI:** 10.3390/jfb14090455

**Published:** 2023-09-03

**Authors:** Sabrina Prete, Marco Dattilo, Francesco Patitucci, Giuseppe Pezzi, Ortensia Ilaria Parisi, Francesco Puoci

**Affiliations:** 1 Department of Pharmacy, Health and Nutritional Sciences, University of Calabria, 87036 Rende, Italy; sabrina.prete@unical.it (S.P.); marco.dattilo@unical.it (M.D.); francesco.patitucci@unical.it (F.P.); gpezzi97@gmail.com (G.P.); francesco.puoci@unical.it (F.P.); 2 Macrofarm s.r.l., c/o Department of Pharmacy, Health and Nutritional Sciences, University of Calabria, 87036 Rende, Italy

**Keywords:** wound healing, wound management, dressings, biopolymers, natural polymers, synthetic polymers, hydrogels, electrospinning, 3D printing

## Abstract

Biomaterials are at the forefront of the future, finding a variety of applications in the biomedical field, especially in wound healing, thanks to their biocompatible and biodegradable properties. Wounds spontaneously try to heal through a series of interconnected processes involving several initiators and mediators such as cytokines, macrophages, and fibroblasts. The combination of biopolymers with wound healing properties may provide opportunities to synthesize matrices that stimulate and trigger target cell responses crucial to the healing process. This review outlines the optimal management and care required for wound treatment with a special focus on biopolymers, drug-delivery systems, and nanotechnologies used for enhanced wound healing applications. Researchers have utilized a range of techniques to produce wound dressings, leading to products with different characteristics. Each method comes with its unique strengths and limitations, which are important to consider. The future trajectory in wound dressing advancement should prioritize economical and eco-friendly methodologies, along with improving the efficacy of constituent materials. The aim of this work is to give researchers the possibility to evaluate the proper materials for wound dressing preparation and to better understand the optimal synthesis conditions as well as the most effective bioactive molecules to load.

## 1. Introduction

Biomaterials exert a significant influence on various stages of wound healing, including cell proliferation, migration, and differentiation, thereby offering innovative avenues for tissue regeneration and repair. Many of these agents exhibit multifunctionality, contributing to different phases of the wound healing process [1]. Among these, synthetic polymer delivery systems stand out as particularly promising materials for tissue regeneration, enabling controlled and sustained drug release [2]. This overview delves into the intricacies of skin regeneration processes and delves into the cutting-edge developments in nanotechnology, highlighting its potential role in the realm of polymeric drug-delivery systems for wound healing, ultimately contributing to the advancement of human health. In this context, drug delivery matrices in this field offer the potential to enhance therapeutic efficacy while simultaneously minimizing risks and adverse reactions [3].

### 1.1. Physiological Native Skin

**Figure 1:**
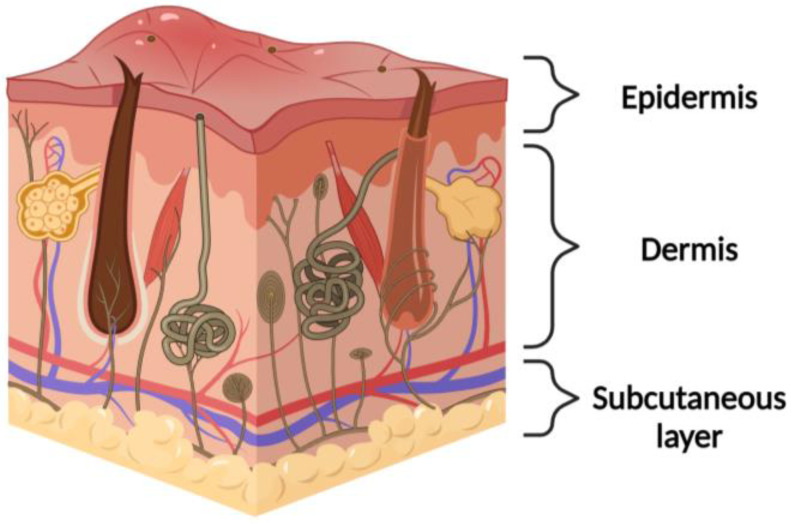
Cross section of the skin: this diagram illustrates the intricate composition of human skin, showcasing the epidermis, dermis, and subcutaneous tissue layers. Key components such as hair follicles, sweat glands, blood vessels, nerve endings, and sensory receptors are also shown, emphasizing their roles in protection, sensation, thermoregulation, and more.

The skin is the largest organ of the body and covers the entire external body surface. It is a continuous tissue, belonging to the integumentary apparatus. It is composed of three main layers that, from the outside towards the inside, are called the epidermis, dermis, and hypodermis (or subcutaneous layer) (Figure 1). The epidermis has a thin and cellular structure that forms the superficial layer of the skin. The basement membrane under the epidermis is the dermis composed of a collagen-rich extracellular matrix (ECM), elastin, fibroblasts, and glycosaminoglycans [4]. Throughout the dermis, there is a network of nerve fibers that has a sensory role in the skin and influences immune and inflammatory responses. The hypodermis is the layer beneath the dermis and contains a large amount of adipose tissue that is well vascularized [5]. The skin has many properties, such as the capacity to self-repair (it regenerates after an injury) and extensibility (it adapts perfectly to the variations in body size that occur throughout life). It provides a defensive barrier against external physical influences such as cold, heat, electricity, and radiation, protecting us from trauma, ultraviolet (UV) radiation, microorganisms, and chemical agents. It prevents the loss of liquids and participates in the mechanism of thermoregulation, exploiting the intervention of the sweat glands and the ability to regulate blood flow, increasing (vasodilation) or slowing down (vasoconstriction) the dispersion of heat [6]. The hypodermis, the deepest layer of the skin, holds significant lipid reserves, contributing to enhanced heat retention in proportion to its lipid thickness [7]. Moreover, the skin has metabolic properties, and the synthesis of vitamin D takes place there. It also has a very important sensory function: with its most superficial layer, it registers and transmits pressure (tactile), pain, and thermal stimuli, while with its deepest layer it also perceives thermal and vibratory signals [8].

### 1.2. Wound Healing Process

The physiological response of an organism to an injury is characterized by a complex mechanism of articulated events, regardless of the type of wound, be it acute or chronic, and the extent of the status of the patient. The healing process of each wound proceeds through many phases that overlap in time and cannot be separated from each other [9]. The process involves the interaction of immune cells (neutrophils, monocytes, macrophages, lymphocytes), non-immune cells (endothelial, fibroblasts, keratinocytes), soluble mediators (cytokines and growth factors), and ECM components [10]. The rate of healing of an acute wound differs from the chronic wound and is dependent on the immunological response [11]. 

**Figure 2:**
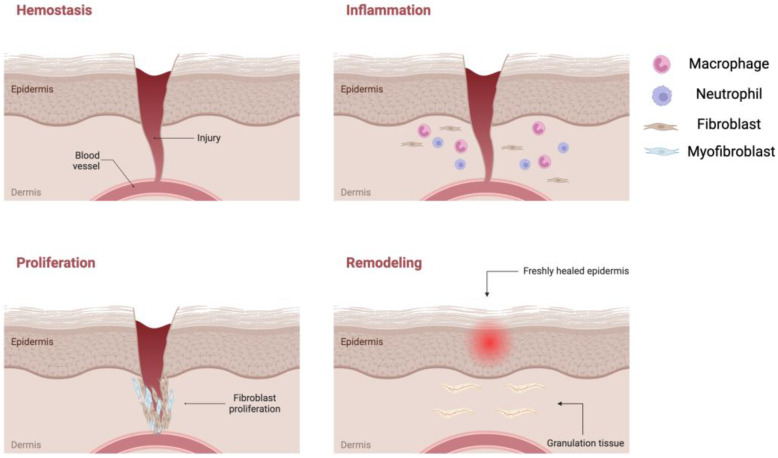
Wound healing phases: (1) hemostasis (the body initiates blood clotting to control bleeding at the wound site), (2) inflammation (white blood cells migrate to the wound to eliminate pathogens and debris, creating an optimal environment for healing), (3) proliferation (new tissue formation occurs as fibroblasts produce collagen, essential for wound strength), (4) remodeling (collagen reorganizes and matures, enhancing tissue strength, and granulation tissue is formed, helping in wound contraction and epithelial cell migration).

Under normal circumstances, wounds heal by themselves following four major phases: hemostasis, inflammation, proliferation, and remodeling (Figure 2) [12].

The initial phase is referred to as hemostasis, during which a newly formed fibrin clot acts as a protective barrier against external elements, ensuring optimal moisture retention [13]. Following hemostasis is the inflammatory stage, where pro-inflammatory cytokines are released from the damaged tissues, attracting circulating white blood cells, and de-granulated platelets and activated macrophages then release various growth factors [14].

The subsequent proliferation phase encompasses crucial processes such as angiogenesis, granulation tissue formation, synthesis of extracellular matrix (ECM) components, epithelialization, and wound contraction [15]. As this phase advances, collagen molecules self-assemble into a distinct triple helical structure and are then released into the extracellular space. Here, they form stable cross-links, imparting strength and stability to the tissue. Dermal collagen typically possesses strong and well-organized properties, whereas scar tissue exhibits smaller and weaker collagen structures. It is important to note that injured tissue never fully regains the characteristics of uninjured skin [16].

**Figure 3:**
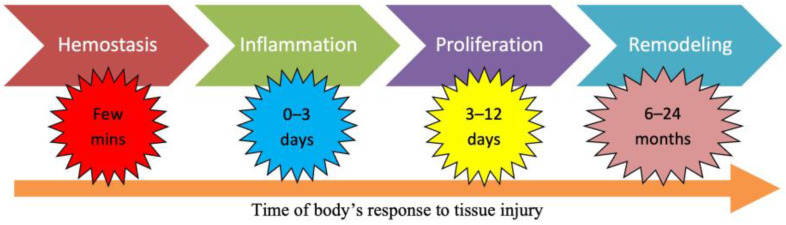
The four phases of acute wound healing immediately proceed for steps from a few mins after an injury to days or months [17].

The concluding phase, known as remodeling, involves the maturation of granulation tissue into scar tissue over a period of 6–12 months (Figure 3) [17]. Excessive granulation tissue formation and an abundance of collagen can result in the development of scars, including hypertrophic scars like keloids [18].

### 1.3. Wound Management

The primary goal of wound management is to facilitate the swiftest possible healing of the wound while minimizing patient discomfort and scarring [19]. Effective wound treatment should aim to minimize scar tissue formation, reduce necrotic tissue production, and thwart the infiltration of microbes. Historically, wound management involved the application of basic gauze materials that did not actively promote the wound healing process. At that time, there was limited consideration for creating an optimal environment conducive to wound healing, or for addressing the functional needs of the wound itself [20]. 

In recent years, different types of medication have been developed, and they are divided into two major categories: traditional dressings and advanced dressings. Traditional dressing refers to a material placed in direct contact with the injured tissue and presents the functions of hemostasis, coverage, and protection, while advanced dressing also aims to maintain a moist microenvironment and constant temperature, remove exudates and necrotic material, protect against exogenous infection, be permeable to oxygen, and reduce trauma on change [21].

The skin’s natural capacity to mend minor injuries is truly remarkable. Yet, in cases of significant damage or when the affected skin area is extensive, the utilization of an appropriate device or dressing becomes crucial. These aids play a pivotal role in safeguarding the wound and expediting the healing trajectory. A wide array of wound management solutions are accessible, spanning diverse physiological forms [22,23].

Wound dressing can be classified according to their nature of action as passive products, interactive products or bioactive products [24].

Gauze and conventional dressings are characterized as passive elements, providing basic coverage. In contrast, polymeric films and foams, distinguished by their transparency, permeability to water vapor and oxygen, and sometimes biodegradability, fall into the category of interactive solutions [25]. Dressings possessing the capability to convey active substances to the wound site are classified as bioactive products. Additionally, specialized devices with distinct attributes, harmoniously blending various constituents in optimized proportions, often involving polymers and hydrocolloids, also play a significant role in wound management [26].

**Figure 4:**
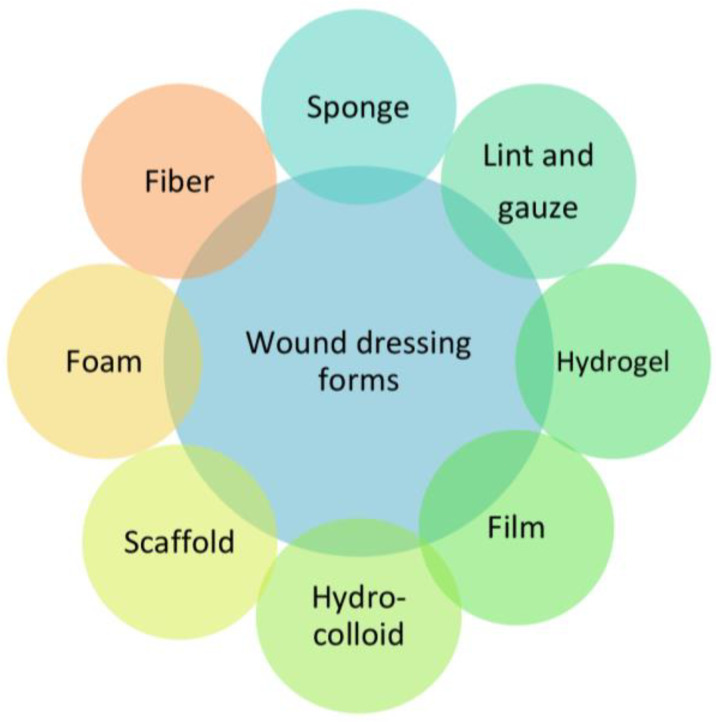
Wound dressing forms.

According to the types and stages of wounds, medical dressings are essential in healthcare. A therapeutic effect is documented for traditional dry dressings (gauze, lint, bandage) which are economical and offer physical protection, but their benefits are limited to the prevention of infection. The current generation of modern dressings (foam, hydrogel, film, scaffold, etc.) offers comfort and many benefits (Figure 4) [27]. 

Ensuring effective wound healing relies on accurately selecting the most suitable materials tailored to the unique requirements of each specific wound [28]. An acute wound is a sudden injury that progresses through the normal stages of healing, including hemostasis, inflammation, proliferation, and remodeling. The healing process typically occurs as expected and follows a predictable timeline. Dressings for acute wounds (hydrogels, foams, or films) often focus on protecting the wound, promoting a moist environment, and preventing infection [29].

Chronic wounds, like venous ulcers or arterial ulcers, are wounds that do not progress or progress slowly through the stages of healing. These wounds often fail to respond to normal healing mechanisms due to underlying issues such as poor circulation, diabetes, or infection [30]. Dressings for chronic wounds aim to address the underlying causes, manage exudate, promote tissue growth, and combat infection to stimulate healing over an extended period [31].

A surgical wound is a wound that occurs as a result of a surgical procedure, such as an incision made by a scalpel or the placement of a surgical drain. These wounds are typically clean and planned. Dressings for surgical wounds focus on preventing infection, securing the wound edges, and maintaining a sterile environment to support healing (post-surgery, sterile adhesive strips or surgical dressings) [32].

A non-surgical wound is any wound that is not a result of a surgical procedure. This category encompasses various types of wounds, including those resulting from trauma, accidents, or underlying medical conditions. Dressings for non-surgical wounds depend on the wound type, depth, and underlying health factors. They can range from simple protective coverings, like sterile gauze or adhesive strips, to more advanced dressings that address infection, exudate, and tissue regeneration [33].

The safety and toxicity of developed wound dressings are critical considerations in their practical application. It is crucial to assess the potential adverse effects or allergic reactions that the dressings might induce in patients. Comprehensive biocompatibility studies should be conducted to ensure that the dressings do not cause irritation, inflammation, or any other undesirable reactions when in contact with the skin. Additionally, the release of any incorporated antimicrobial agents or other active substances should be evaluated to ensure that they remain within safe limits and do not lead to toxicity.

Moreover, the degradation products of the dressings, if any, should be investigated for their potential impact on the surrounding tissues. The choice of materials, cross-linkers, and additives in the dressing formulation can significantly influence its biocompatibility and toxicity. Ultimately, rigorous testing and adherence to regulatory guidelines are essential to ensure that the developed wound dressings are safe, effective, and suitable for clinical use [34,35].

### 1.4. Traditional Dressings

Although gauze dressings have been the standard treatment for many wounds for decades, this treatment method is now outdated. It has many negative impacts on the healing environment and leads to increased pain and suffering for the patient. Gauze and cotton gauze composites have very high absorption capacity, and they are generally used during the first intervention in a wound treatment by direct contact. As they cause rapid dehydration, when they are being removed from the wound surface, they can cause bleeding and damage to newly formed epithelium [36]. Therefore, there are prepared gauze composites with a non-adhesive inner surface able to reduce the pain and trauma caused by gauze removal. One of the main problems of traditional dressing materials consists of the risk of infection, so antibacterial agents can be added into the dressings as a prevention method (e.g., paraffin gauze dressing containing 0.5% chlorhexidine acetate or 3% bismuth tri bromo phenate) [22,37].

Chlorhexidine is an antimicrobial agent that is active against a wide range of Gram-positive and Gram-negative bacteria [38]. It is non-adherent, non-allergenic, gamma sterile, and reduces the adherence of the product to the surface of a wound [39].

Paraffin can also reduce adherence to the wound surface and works to soothe the injury [40]. Paraffin gauze dressings are ideal for burns and wounds of medium identity and superficial skin loss. They protect the wound, allowing drainage onto a secondary absorbent dressing [41]. One of the most significant problems encountered by using this material is a foreign body reaction in the wound caused by cotton fibers [42]. Clinical signs more frequently observed after gauze and tape dressing use are skin maceration and hyperemia; the biggest advantage is their low cost and the possibility of being formulated in many forms as tapes, rolls, or adhesive patches [43]. 

## 2. Biomaterial-Based Dressings

Employing biomaterials in wound care offers a multitude of advantages stemming from their exceptional characteristics such as biocompatibility, capacity to foster cell growth, regenerative potential, biodegradability, and durability. These biomaterials encompass both natural and synthetic polymers, harnessing the benefits of both domains [44,45].

### 2.1. Natural Polymers

**Figure 5:**
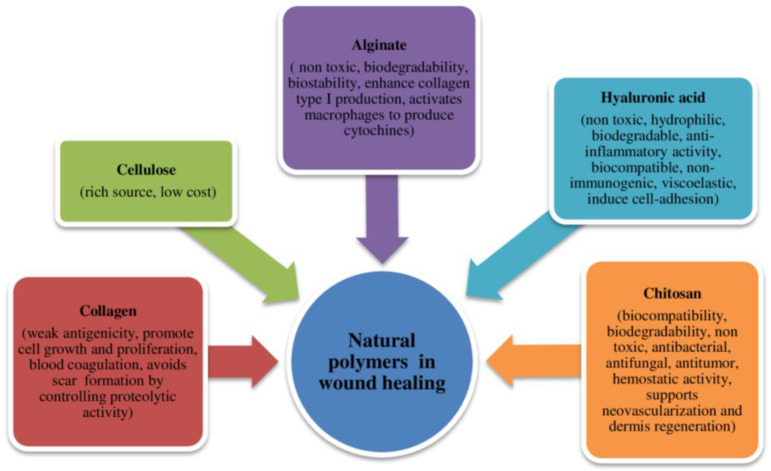
Some functional biomaterials identified as promising wound healing applications and their main characteristics.

Natural polymers, also known as biopolymers, are organic compounds synthesized by living organisms. These molecules have a structural arrangement characterized by sequences of repeating units or monomers, typically amino acids or monosaccharides, which combine to form peptides and polysaccharides. Biopolymers originate from various sources, including plants, animals, fungi, bacteria, and algae. Their diverse origins make them applicable across numerous fields due to their distinct properties (Figure 5) [46]. The natural source of these polymers positions them as optimal substitutes for the extracellular matrix (ECM) and the original cellular environment of native skin. Natural polymers offer several advantages over synthetic materials, including high biocompatibility, biodegradability, reduced antigenicity, and renewability [47].

#### 2.1.1. Collagen

Collagen, the primary connective tissue protein in animals, imparts tensile strength to the skin and plays a pivotal role throughout all stages of wound healing. Its influence extends to attracting crucial cells like fibroblasts and keratinocytes to the injury site, thereby promoting angiogenesis and facilitating re-epithelialization. By furnishing an indispensable physical framework, collagen establishes a vital scaffold for new tissue growth. Chemically, collagen is composed of triple-helix fibrils, with each fibril constituting a polymer of recurring amino acids interconnected by peptide linkages. It exists in both its native form and a denatured state known as gelatin [48]. In the inflammatory phase, various proteolytic enzymes are released, cleaving collagen into smaller fragments. These fragments contain Arg-Gly-Asp (RDG) sequences that act as chemoattractants for macrophages and mitogens for fibroblasts, thereby inducing granulation and stimulating new tissue proliferation within the wound bed [49,50]. These dressings possess the ability to chemically bind matrix metalloproteinases (MMPs) present in the extracellular fluid of wounds. Ordinarily, MMPs degrade collagen, yet the use of collagen-based dressings provides an alternative attachment site for MMPs, thus preserving the body’s natural collagen for customary wound healing. Gelatin is formed through collagen cleavage and, alongside inherent collagen, it curtails excessive MMP activity, allowing the proliferation phase to dominate over inflammation. Augmenting the physical, mechanical, and functional attributes of biopolymers can be achieved by constructing scaffolds and amalgamating them with other molecules [51].

Collagen finds application in the development of delivery systems such as nanofibers, nanoparticles (NPs), hydrogels, films, and sponges that aid in the healing process [52].

Presently, collagen is used in numerous biomedical applications as a suspension for dermal injections, hemostatic agents (collagen sponges), wound dressing materials, collagen sutures and catguts, collagen gels for periodontal reconstruction, and for joint coating. 

Collagen is an excellent wound dressing material because it has demonstrated excellent biocompatibility and mechanical stability. Collagen dressings are easy to apply and remove. The ones formulated from bovine, porcine, or avian sources are recommended for the treatment of partial- and full-thickness wounds with minimal to moderate exudates. It is contraindicated for third-degree skin burns and for sensitive/allergic patients [53]. In recent years, many studies reported the use of collagen for the release of drugs such as antibiotics. Puoci et al. developed a conjugate based on collagen and fluoroquinolone as an antibacterial in wound healing. The antibiotic ciprofloxacin was covalently bonded to the protein and its local administration is used for diabetic foot treatment. This strategy highlighted the use of collagen as a biomaterial to promote fibroblast growth [54]. 

In another work, quercetin and curcumin were separately incorporated in different experimental set-ups into a collagen matrix, showing healing properties on a diabetic wound by effective scavenging and re-epithelialization [55,56].

Numerous studies have been carried out concerning the application of different collagen dressing formulations for wounds and burns [57,58,59,60,61,62,63,64]. Moreover, collagen nanofibers produced by the electrospinning technique have promoted better wound healing than other fabrication methods [65]. 

#### 2.1.2. Cellulose

Cellulose, the most abundant biopolymer found in plant cell walls, is a polysaccharide comprising repeating units of β-D-glucose interconnected by β-1,4 bonds. Notably, certain bacteria, including those of the genera Acetobacter, Sarcina ventriculi, and Agrobacterium, also produce cellulose, with bacterial cellulose standing out for its relatively high purity [66]. Bacterial cellulose holds considerable promise as a biopolymer due to its capacity to manage wound exudate and create a conducive moist environment for wound healing. However, its application is hampered by a lack of inherent antibacterial activity. The nanostructure of cellulose lends itself to advantageous physicochemical and mechanical properties, alongside biocompatibility and biodegradability. Cellulose finds applications due to its intricate porous 3D structure, which mimics the extracellular matrix (ECM) of the skin, thereby fostering tissue regeneration [67,68,69]. Bacterial cellulose presents potential across various domains, including drug delivery, bioprinting, implants, and artificial organs [69]. Cellulose can be effectively blended with other materials, including antimicrobials, to counteract infections at wound sites. Incorporation of cellulose with agents like lysostaphin [70], silver NPs (AgNPs) [71], and silver sulfadiazine [72] has demonstrated bactericidal properties against *E. coli* and *S. aureus*. Notably, Lin et al. reported that cellulose membranes derived from bacteria and coated with chitosan facilitated enhanced epithelialization and expedited tissue regeneration [73]. New polyvinyl-alcohol/carboxy-methylcellulose (PVA/CMC) blend composite films were formulated to take advantage of the combined properties of both polymers—swelling capacity, elasticity, water solubility, porosity, water vapor transmission rate, and biodegradability—for the tissue repair process [74]. The hydrophilic nature of CMC makes it possible to mix and cross-link with other materials, such as synthetic polymers, natural polymers, and inorganic materials, and enables the preparation of innovative biomaterials for wound dressing. In addition, CMC dressings are used for their flexibility, ability to absorb exudate, promotion of angiogenesis, and autolytic debridement [75,76,77,78]. In a separate study, a hydrogel was created through UV cross-linking of cellulose and acrylic acid, resulting in an innovative material that exhibited enhanced neovascularization and re-epithelialization in burn wound healing [79].

Diaz-Gomez et al. fabricated a CMC scaffold by 3D printing, which was then used for the treatment of diabetic wounds. The scaffolds loaded with platelet-rich plasma showed a release of growth factors and promoted angiogenesis, granulation, and re-epithelization [67]. Cellulose activity was assessed at different pH values and acidic cellulose showed improved healing properties in a cutaneous wound model [78]. 

In the study by Shin et al., a novel approach was undertaken to enhance the properties of a hydrogel. This involved combining sodium carboxymethyl cellulose (NaCMC) with PVA and polyethylene glycol 400 (PEG 400) using a cyclic freezing/thawing technique. As a result of this innovative method, a hydrogel was developed that exhibited notable enhancements in various aspects: increased swelling rate, heightened compressive strength, and improved cytocompatibility. The hydrogels that were synthesized exhibited a porous architecture, which contributed to their commendable swelling capacity. Furthermore, these hydrogels demonstrated favorable cellular proliferation and played a role in fostering the wound healing process [80,81]. 

In a different investigation, a bi-layer dressing hydrogel was developed using a combination of PVA, CMC, and PEG via a thawing/freezing technique. The process involved pouring the polymer solution into a template, allowing it to stand in a mold for 35 min, and subsequently placing it in a refrigerator at −20 °C for 20 h to form the upper layer, characterized by small pores measuring less than 20 µm. This bi-layer dressing demonstrated favorable mechanical properties, effectively hindered bacterial penetration, and regulated wound moisture to sustain a moist environment. Notably, the bi-layer hydrogel facilitated an accelerated wound closure process [82].

#### 2.1.3. Chitin and Chitosan

Chitosan is a copolymer composed of N-acetyl-2-D-glucosamine and 2-D-glucosamine, generated through partial deacetylation of chitin. Chitin, the second most abundant biopolymer in nature after cellulose, predominantly resides in the exoskeleton of arthropods and the cell walls of fungi. The physical and chemical attributes of chitosan, encompassing water solubility, biodegradability, and reactivity, hinge on the quantity of free amino groups within the chain, which correlates with the degree of acetylation of the polysaccharide. While chitin is generally insoluble, it can undergo deacetylation to yield the soluble polymer chitosan. The amino groups within chitosan engage with a multitude of negatively charged proteins and glycolipids present on the exterior of red blood cells (RBCs) [83]. This interaction leads to heightened blood viscosity, instigates platelet adhesion, and substantially contributes to its hemostatic function. Furthermore, the antimicrobial attribute of chitosan has been linked to its inherent cationic character [84].

Chitin and chitosan serve as noteworthy biopolymers recommended for innovative biomatrix applications, including drug delivery devices, tissue engineering scaffolds, and bioactive dressings. Their notable attributes include their non-toxic and non-immunogenic characteristics, excellent biodegradability, antimicrobial activity, and the ability to expedite wound healing. Thanks to these advantageous intrinsic qualities and their high potential for fostering wound healing, these compounds stand as appealing biopolymers for wound management. Chitosan, in particular, is employed as a functional material for wound treatment owing to its hemostatic effects during the initial stages, its capacity to inhibit microbial growth, and its capability to accelerate wound healing. Chitosan can take various forms, such as membranes, hydrogels, fibers, and sponges [85].

Carboxymethyl chitosan represents a chemical modification of chitosan formed by attaching carboxymethyl groups to its backbone. Carboxymethyl chitosan can significantly promote the proliferation of the normal skin fibroblasts, but it also inhibits the proliferation of keloid fibroblasts [86]. It has the advantage of a greater solubility range compared to native chitosan and has now been extensively studied for drug delivery and for its properties of hemostasis, antimicrobic activity, and the stimulation of wound healing. Different formulations of wound dressing containing chitin and chitosan have been developed. Chitin/chitosan hydrogels or composite membranes have been formulated adding zinc oxide NPs (ZnO-NPs) [87]. Films, sponges, and other hydrogels have also been formulated with microcrystalline chitosan and antibacterial AgNPs [88]. Chitosan films have been enriched with antioxidant agents such as diosgenin or aloe vera, two substances relevant to wound injury treatment [89,90]. Other films incorporating thyme, clove, and cinnamon oils based on chitosan have been prepared for potential wound healing applications and for their antimicrobial activity against Gram-positive and Gram-negative bacteria [91].

Recent studies showed lipid nanocarriers containing active antimicrobial agents, such as chlorhexidine, incorporated into chitosan hydrogel. These vesicles called *Chitosomes* incorporated in the hydrogel showed a better antimicrobial effect against *S. aureus* and *S. epidermidis* compared to the formulations without chitosan. This novel product could be used for infection prevention and bacterial eradication in acute wounds [92].

#### 2.1.4. Alginate

In the pharmaceutical field, alginate is produced from brown algae cell walls and from some bacteria strains such as *Pseudomonas* or *Azobacter*, and then it is harvested, dried, chemically processed to remove impurities, and transformed into powder in the form of a salt (sodium alginate).

The chemical structure of alginate is composed of α-L-glucuronic acid (G) and β-D-mannuronic acid (M) linearly linked by 1,4-glycosidic linkages. The sugar units are distributed in blocks or alternated, and their distribution depends on the source from which the material came from. The molecular weight of sodium alginate varies between 32,000 and 400,000 g/mol and the solubility depends on the chemical composition; if alginate is rich in glucuronic acid, it will be more soluble in water than if it is rich in mannuronic acid. On the other hand, the viscosity of the alginate depends on its concentration, molecular weight, and the length and number of monomers units; the longer the length of the segments, the higher the viscosity will be [93]. Alginate is used in wound healing for its ability to reduce local pain, its cooling effect, and its property of non-adhesion to the wound bed. The presence of di- and trivalent ions in the water initiates the formation of a gel. An alginate-containing dressing in contact with wound exudates initiates the ion exchange between calcium ions in the alginate and sodium ions in blood or exudates. When sufficient calcium ions are replaced by sodium ions, the alginate fibers partially swell, dissolve, and form the gel [94]. This property represents the basis for the biological and industrial applications of alginate. In the pharmaceutical field, this compound is used in ulcer repair processes because it has the property of being highly absorptive and can reach up to twenty times its weight [95]. This characteristic depends on its composition; if the glucuronic acid content is high, the dressing will have a higher absorptive capacity. Alginate intervenes in the process of exudate absorption to create a favorable ulcer microenvironment to promote epithelization [93]. For these characteristics, alginate dressings are suitable for ulcers with high exudate production [95] and also shows good hemostatic properties [96]. If calcium alginate is applied to the wound and left for 5 min, its hemostatic activity can be demonstrated [97]. Alginate is biocompatible and, therefore, does not cause systemic reactions following administration and is biodegradable. When in contact with body fluids, it breaks down into monosaccharide residues which are completely absorbed and the conversion to a sodium salt facilitates the removal of residues by dissolution. Highly porous 3D calcium alginate scaffolds exhibit exceptional swelling capabilities within wounds, facilitating gradual drug release. These scaffolds find application in entrapping cells for the purpose of tissue regeneration and engineering [98,99]. Alginate finds prominent application as hydrophilic hydrogels, capitalizing on its ability to absorb substantial quantities of water and thereby safeguard the wound bed against desiccation. In recent developments, thermosensitive hydrogels have been synthesized utilizing alginate and poly(N-isopropyl acrylamide) as the polymer network, incorporating them onto cotton fabrics. This innovation facilitates drug release through modulation of the thermosensitive hydrogel layer’s thickness, offering a versatile mechanism for controlled drug delivery [100].

Recently, alginate microspheres, as well as combinations such as alginate–chitosan and alginate–chitosan–chito-oligosaccharide scaffolds, have been effectively utilized as substitutes for skin. These scaffolds have demonstrated heightened biocompatibility, closely mimicking the natural skin microenvironment. Remarkably, their implementation has led to a rapid wound healing rate exceeding 90% within a mere 14-day timeframe [101].

To promote new tissue formation, curcumin-loaded NPs have been used for skin damage because of their low toxicity and anti-inflammatory and antibacterial properties. Due to their low water solubility and low oral absorption, clinical applications are limited to inside NPs or micelles. Fourier-transform infrared spectroscopy (FT-IR) studies indicated that curcumin NPs were properly embedded in the alginate/collagen scaffold leading to an improved healing process; curcumin has the effect of anti-scar formation and reduces the secretion of inflammatory factors [102].

Calcium-alginate-based materials play a crucial role in the final stages of wound healing. When using chitosan alone, the resulting film is notably rigid, necessitating the incorporation of plasticizers to render it suitable as a flexible wound dressing material. However, by introducing sodium alginate to chitosan, the polymer matrix’s flexibility can be significantly enhanced. This combination can undergo cross-linking facilitated by divalent cations like Ca^2+^, resulting in the formation of stable, biodegradable gels that are well-suited for cell encapsulation and immobilization. Numerous studies have explored the development of a bio-composite matrix to expedite wound healing, facilitating the regeneration of essential cells like fibroblasts and keratinocytes. As an illustration, a chitosan–alginate fibrous matrix has been shown to facilitate the release of Ca^2+^ ions from incorporated calcium phosphate (CaP-NPs) and zinc oxide NPs (ZnO-NPs). The presence of Ca^2+^ ions accelerates cell proliferation, while the synergistic effect of chitosan and ZnO-NPs provides an effective defense against bacterial invasion within the wound bed [103].

Infection during the wound healing process remains a critical threat which can lead to severe complications. Tao et al. realized a matrix of alginates with immobilized hydroxyapatite and zinc oxide in the presence of metal ions and chitosan as crosslinkers, stimulating collagen deposition in the infected wound and promoting skin reconstruction. The presence of metal ions lowered the pH of the environment, promoting the healing process. The combination of Ca^2+^, Zn^2+^, and Cu^2+^ with chitosan increased the antimicrobial effects against *E. coli* and *S. aureus* [104].

#### 2.1.5. Hyaluronic Acid

Hyaluronic acid (HA) is the main component of ECM; it promotes cell migration thanks to its enormous capacity to bind water, which facilitates cell movement. It was discovered in the vitreous eyes of the cows by Karl Meyer and John Palmer. Subsequent studies showed that it is present in all vertebrate animals and human beings. HA generates an optimal microenvironment that stimulates the increase, proliferation, and migration of fibroblasts, endothelial cells, and keratinocytes, and promotes neo-angiogenesis. In the proliferative phase of the tissue repair process, granulation tissue is formed through the action of HA, fibronectin, laminin, collagen, and other glycosaminoglycans. HA is a natural and linear polysaccharide, consisting of repetitive units of D-glucuronic acid and N-acetyl-D-glucosamine bound by β (1 → 3) and β (1 → 4) glycosidic linkages. HA properties depend on its molecular size. It has also antioxidant activity and activates a series of cascades by binding CD44 receptors present on the keratinocytes, facilitating differentiation [105]. Examples of wound care commercial products derived from HA are Dermaplex, Hyalomatrix, and Hyalofill. HA has been widely used for the treatment of acute and chronic skin lesions, burns, ulcers, pressure sores, and diabetic foot. It is known by its versatility and researchers have modified the polymeric backbone with different chemical groups in order to improve its mechanical, rheological, and swelling properties [106].

HA scaffolds have demonstrated a notably potent hemostatic effect in comparison to other wound dressings. HA scaffolds serve as stable carriers for cells and hold the potential to promote tissue volume retention. Various derivatives of HA have been developed through functional group modifications, enhancing its properties and rendering it suitable for medical applications. 

Given that HA is naturally produced by cells during the initial stages of wound healing, it has garnered significant attention for wound dressing applications. HA plays a pivotal role in the angiogenic phase of wound healing by modulating inflammation at the wound site, acting as a scavenger for free radicals, and interacting with receptors on diverse cells associated with tissue repair [107]. The angiogenic impact of short-chain HA molecules, ranging from 3 to 10 disaccharide units, has been observed to be regulated through two receptors: the receptor for HA-mediated motility (RHAMM) and CD44. These receptors are associated with the proliferation and migration of endothelial cells during the healing process [108].

In a study conducted by Makvandi et al., a distinctive approach was taken to produce thermosensitive and injectable hydrogels utilizing HA in combination with corn silk extract (CSE) and AgNPs. These hydrogels demonstrated the capability to exhibit antibacterial activity against both Gram-positive (*B. subtilis*, *S. aureus*) and Gram-negative (*P. aeruginosa*, *E. coli*) bacteria [109].

Recently, antimicrobial peptides (AMPs) have gained recognition as a promising category of antimicrobial agents and potential candidates for new antimicrobial drugs, offering a potential solution in the battle against antibiotic resistance. Notably, a specific AMP called Tet213A, when incorporated into alginate substrates along with HA and collagen, exhibited remarkable antimicrobial effects against *E. coli*, methicillin-resistant *S. aureus (MRSA)*, and *S. aureus*. This resulted in the inhibition or eradication of bacteria within infected wounds. The prompt release of AMP from these wound dressings facilitated effective healing, fostering collagen deposition and promoting angiogenesis [110].

### 2.2. Synthetic Polymers

The class of synthetic polymers includes those polymers that are biocompatible, bioresorbable, and, being synthetic, have reproducible properties that can be adapted to a given application.

**Figure 6:**
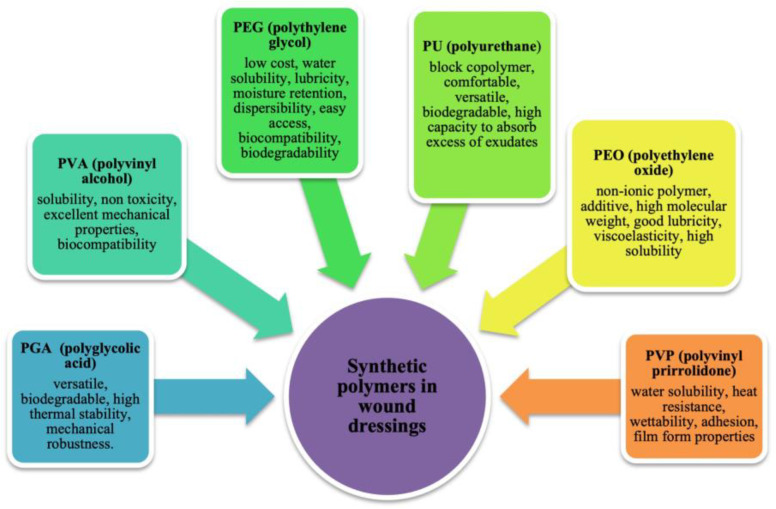
Schematic properties of the main synthetic polymers used in wound healing.

The difference between synthetic and natural polymers is that the former can be synthesized and modified in a controlled manner to achieve specific properties or stability. This type of polymers includes PVA, polyethylene oxide (PEO), PEG, poly(ε-caprolactone) (PCL), polyurethane (PU), poly lactic acid (PLA), poly vinyl pyrrolidone (PVP), and polyglycolic acid (PGA) (Figure 6) [111]. The physical, chemical, mechanical, and kinetic characteristics of these materials, unlike natural materials, can be adapted to the type of desired application. Some of the main disadvantages of synthetic polymers include the high cost to use and sometimes have a very different structure from the extracellular matrix [112]. It is possible to functionalize the material to give it the property of bioactivity by immobilizing biomolecules within the structure or on its surface, such as peptide sequences, adhesion proteins, or polysaccharides [113]. Alternatively, blends can be made by mixing synthetic materials with a natural polymer or with biomolecules to create a complex (Interpolymer complex) [114].

#### 2.2.1. Poly-Vinyl Alcohol (PVA)

PVA is a water-soluble synthetic polymer, mainly composed of 1,3-diol linkages. It is prepared by hydrolysis of polyvinyl acetate. PVA is a common polymer with good solubility and excellent mechanical properties, and it is used in biomedicine for its biodegradability, low toxicity, and biocompatibility. However, pure PVA hydrogels do not present hemostatic and antibacterial effects and they lack elasticity and hydrophilicity [74,115,116]. In recent times, researchers have directed their attention toward synergizing PVA with additional functional constituents, including chitosan, gelatin, oxidized cellulose, as a means to enhance wound healing [117]. A recent study showed PVA being used in matrix development in combination to other composite materials or bioactive substances and its interaction with cells accelerates the wound healing process [118].

Hydrogels of PVA/Pullulan/Poly-L-Lysine/Gelatin enhanced wound healing, exhibiting low toxicity in vitro and better blood compatibility thus improving cell migration and proliferation [119].

The synthesis of hydrogels incorporating PVA, dextran, and chitosan has exhibited promising wound healing properties for burns or decubital ulcers. A study conducted by Lin et al. showcased the combination of PVA, dextran, and chitosan, forming an optimal wound dressing material cross-linked using glutaraldehyde (GA). This polymeric matrix not only demonstrated antimicrobial efficacy but also exhibited enhanced thermostability, water retention, mechanical properties, and moisturizing capabilities within the PVA hydrogel. In summary, the PVA/chitosan/dextran hydrogel has displayed considerable potential as a promising candidate for advanced wound healing applications [120]. 

Hydrogels composed of PVA and aloe vera have been synthesized using a cross-linking method involving propanol to enhance the crystalline structure of PVA. In order to enhance the biological properties of the hydrogel, curcumin was incorporated, while gentamicin was added to harness its antimicrobial capabilities. Results showed that aloe vera has a high impact on gentamicin release in wound zone.

Histological evaluations exhibited a heightened rate of re-epithelialization in comparison to wounds treated with cotton gauze or PVA/aloe vera dressings devoid of curcumin. This underscores the potential of PVA/aloe vera hydrogels, especially when supplemented with curcumin and gentamicin, to positively impact wound healing and re-epithelialization processes [121].

#### 2.2.2. Polyethylene Oxide (PEO)

PEO and PEG refer to the same polymer, but they are different in molecular weight: PEO is available in high molecular weights, while PEG cover the lower molecular weights range. They are non-ionic polymers used in electrospinning or as additive with other polymers [122]. PEO presents high solubility in an aqueous environment, good lubricity, and viscoelasticity [123]. Many papers describe the application of chitosan with PEO. In particular, they showed its in vitro capacity to absorb the exudate [124].

Szymanska et al. developed a porous nanomaterial composed of PEO and medical-grade chitosan, ingeniously integrated within fibers. When exposed to simulated wound exudate, this concoction assumed a gel-like structure, essential for the facilitation of the healing process. In meticulous in vitro studies centered around wound exudate absorption, this formulation showcased promising potential as a dressing suitable for wounds characterized by low to moderate exudate levels. Remarkably, this material exhibited reduced adhesion to excised human skin surfaces following a coating of poly(dimethyl siloxane). Furthermore, comprehensive tests involving contact with EpiDerm™ tissue substantiated the material’s non-irritating nature, confirming its compatibility with a simulated skin model. In addition, through an in vitro scratch assay, the material’s capacity to stimulate fibroblast migration, even in the early stages of wound healing, was strikingly demonstrated [123].

#### 2.2.3. Polyethylene Glycol (PEG)

PEG is an amphiphilic polymer composed of monomer units of oxide ethylene; its properties mainly depend on molecular weight. It is biocompatible, biodegradable, and hydrophobic and it is widely used in biomedical field. PEGs are non-toxic, generally non-immunogenic, and they are used for drug delivery systems; their surface can be functionalized and can be used in tissue engineering. PEGylation is the covalent conjugation of drug targets such as peptides, proteins, or oligonucleotides with the polymers for the optimization of pharmacokinetic properties [125]. PEG is used in hydrogel preparation for tissue engineering and, in recent years, also in wound dressings because of its non-toxicity, good biocompatibility, biodegradability, easy availability, stable activity, and low preparation cost. Nevertheless, the incorporation of cross-linking agents has introduced cytotoxicity concerns for the resulting hydrogel dressings. Consequently, contemporary researchers have shifted their focus toward mitigating the toxicity associated with PEG-based hydrogels. A noteworthy illustration of this effort lies in the adoption of citric acid (CA) as an alternative cross-linking agent. CA has been employed to craft hydrogels aimed at fostering the healing of chronic wounds, offering a potential solution to the cytotoxicity issue [110]. 

#### 2.2.4. Polyvinylpyrrolidone (PVP)

PVP boasts attributes like low toxicity, excellent water solubility, biocompatibility, biodegradability, heat resistance, wettability, adhesion, and the ability to form films. Its versatile nature finds applications across domains such as medicine, food, cosmetics, and beyond [126]. In recent years, the permeability of PVP to bacteria has spurred the widespread adoption of PVP-based hydrogel dressings. Notably, Khan et al. devised hydrogels by incorporating Ag@ZnO nanocomposites (NCs) within PVP/PVA matrices. The resultant dressing exhibited antibacterial properties, effectively curbing infection rates and thereby accelerating the wound healing process. The NCs showed remarkable activity towards *S. aureus* and *MRSA*. The potential antibacterial mechanism was found to be reactive oxygen species (ROS)-dependent. PVP/PVA/AgZnO NCs hydrogels have been compared to PVP/PVA hydrogels; the data suggested that the antibacterial activity is enhanced with the increase in NC concentration after 6 h of incubation [127].

To create an appropriate microenvironment for the healing process, it is also possible to utilize spun PVP as nanofibers able to mimic the fibrous extracellular matrix, support neo-tissue growth, and release drugs in the wound bed. Moydeen et al. synthesized nanofibers of PVP and dextran sulfate as a drug delivery system with a single-nozzle core shell. PVP/dextran sulfate nanofibers loaded with ciprofloxacin showed a broad antibacterial activity that was evaluated using the disc diffusion method against different Gram-positive wounds (*MRSA, S. epidermidis*, *S. aureus* and *Klebsiella pneumoniae*) and Gram-negative bacteria (*Pseudomonas aeruginosa*, *Salmonella typhimurium* and *Proteus vulgaris*), and in vitro studies revealed that the release mechanism mainly depended on diffusion [128]. 

#### 2.2.5. Polyurethane (PU)

PU is a block copolymer produced through the reaction of a diisocyanate with a polyol. Conventional polyols are polyethers or polyesters, where the polyol segment provides a low glass transition temperature (i.e., <25 °C) and represents the soft segment, while the diisocyanate, often combined with a hydrocarbon chain extender, provides the hard segment. PU is the most versatile polymer because it can be produced as resin, plastic, elastomer, or in adhesive form, with the choice of structure and composition of the components under appropriate reaction conditions [129]. 

Traditional aromatic diisocyanate compounds are potentially carcinogenic, so biodegradable polymers with nontoxic degradation products have been made from diisocyanates, such as lysine-diisocyanate or hexamethylene-diisocyanate [130].

For biodegradable polyurethane production, poly (α-hydroxy)acids have been used as soft segments, including PLA, PGA, and PCL [131].

PU as a wound dressing is usually used as foam or film, able to maintain an optimal moist microenvironment for wound healing. PU dressings have been applied to a wide range of wounds: decubitus ulcers, granulating wounds, chronic, and acute [132].

PU-Gel nanofibrous membranes, loaded with honey and ZnO-NPs, showed antibacterial activity against *S. aureus*, *Escherichia coli*, and *Bacillus subtilis* [133].

The use of traditional PU foams has been restricted to bio-clinical applications because of the toxicity of their petroleum-originated preparation and bioinert nature, but it has been proven to be clinically valuable for wound treatment [134]. In order to replace petroleum products and support a circular economy, a friendly PU material has been developed for biomedical applications. For example, NPs of PU and ions of Ag have been prepared using the biomass derived from raw materials. Lignin and its derivatives, consisting of abundant aromatic groups, represent biomass materials derived from agricultural raw materials and showed antibacterial activity [135].

#### 2.2.6. PGA (Polyglycolic Acid)

PGA or polyglycolide is a biodegradable thermoplastic polymer, the simplest linear aliphatic polyester. It can be synthesized by condensation or ring-opening polymerization of glycolic acid. PGA has been known since 1954 as a polymer capable of forming strong fibers [136], but because of its hydrolytic instability its use has been limited. PGA is a biodegradable resin that offers high mechanical strength and high gas barrier performance. Because of its high molecular weight, PGA can be produced at a lower cost than before, and several applications have been developed to take advantage of its characteristics [137].

Recently, PGA has been used in wound healing since some studies suggested that it plays a role in the neo-epithelium formation and inflammation processes [138]. PGA has found applications in preventing delayed perforation and in the closure of a fistula in open and endoscopic surgery or to cover wounds following partial glossectomy [139]. An open wound surface, too, has been covered with PGA and fibrin glue. PGA sheets are an absorbent strong material, but they are gradually degraded by hydrolysis. Fibrin glue is a biodegradable and absorbable biological agent used in tissue repair with hemostatic capacities to prevent liquid or air leakage. The utility and application of these adhesives have been previously reported [140].

## 3. Classification of the Dressings by Physical Form

### 3.1. Bandages

Conventional approaches to wound treatment involve the utilization of bandages and gauze, serving as materials to absorb exudates and offer physical shielding. These dressings are inherently dry and lack the capacity to maintain a moist environment. As the fluid content decreases, they tend to adhere to the wound site, resulting in discomfort and pain when removal is attempted [141].

### 3.2. Lyophilized Wafers

Freeze-dried wafers are created by transforming polymeric solutions or gels into solid, porous structures. These wafers possess the unique ability to incorporate drugs within their matrix. They exhibit a remarkable capacity to absorb fluids, including excessive exudates, while also facilitating the gradual diffusion of the drug within the wound bed [142].

### 3.3. Hydrogels

Hydrogels are insoluble polymers in water, which creates a hydrophobic crosslinked network. They are highly absorbent and do not lose their network structure; they can donate water molecules and maintain a moist environment at the wound bed. Hydrogels transmit moisture vapor and oxygen. Moreover, they are non-toxic, biocompatible, and reversible for medical application. They promote wound debridement by rehydration of non-viable tissue, thus facilitating the process of natural autolysis. Hydrogels have been used as standard form in the management of necrotic wounds. They are not indicated for wounds with high levels of exudate or in the presence of gangrenous tissue, which should be kept dry to reduce the risk of infection [143]. Hydrogels also possess a degree of flexibility similar to tissue and offer the advantage of incorporating many bioactive agents in a specific space, thanks to their tridimensional structure, and releasing them in the environment, usually by a gel-sol transition to the liquid state [110,144]. 

### 3.4. Films

Film polymers may include co-polymers, homopolymers, and plasticized polymers consisting of a series of sheets, like sheets of PU coated with hypoallergenic acrylic adhesive. They should be impermeable to fluids and bacteria and permeable to air and water vapor. Through this mechanism, dressings are able to create a moist wound environment. In wound healing, they can be used as primary or secondary dressing or can be incorporated into hydrogels or foams to create composite dressing [145]. In the past, dressing films were usually made of nylon and supported in an adhesive polyethylene frame, but they were much occlusive. Modern dressing films are semi-permeable adhesive sheets, very flexible, and they are good for wounds but also over joints [56]. However, they are unable to cope with large amounts of exudate and may cause maceration of the skin surrounding the wound bed if they are used injudiciously. Films are used for superficial wounds and wounds with light exudates [144].

### 3.5. Patches 

Wound patches have shown immense potential in offering properties such as ultra-adhesion, self-healing capabilities, biosensing functionality, antibacterial effects, and anti-inflammatory attributes. Recent times have witnessed a significant surge of interest in wearable patches, driven by their distinct advantages including flexibility, non-invasiveness, real-time monitoring, seamless integration, sensitivity, and robust stability. As the demand for personalized medical care continues to rise, wearable patches have emerged as a focal point due to their substantial potential in shaping the next phase of wound management [146]. These bio-sensorial patches rely on biochemical and physiological sensing mechanisms, which encompass parameters like pH variation, glucose levels, and temperature shifts. For instance, unhealed wounds typically exhibit an alkaline pH compared to the skin’s acidic pH. Additionally, the concentration of blood glucose in a wound serves as not only an indicator of a diabetic patient’s physical state but also influences the extent of bacterial infection within the wound.

The fabrication of these patches often involves 3D printing technology. The selection of an appropriate bioink is of paramount importance in the production of 3D-printed patches. This bioink must exhibit high biocompatibility, mechanical stability, and exceptional post-printing shape fidelity. As wearable patches continue to evolve, they hold significant promise for revolutionizing wound management and ushering in a new era of personalized healthcare [147].

### 3.6. Scaffolds

In the last few years, researchers have included biomaterials in the creation of 3D scaffolds. Deep wounds are unable to regenerate themselves, so the development of specific scaffolds is essential to promote the natural sequence of healing events by providing mechanical support for the growth of new tissue. A scaffold is a structural framework that provides support and cohesion to living tissue. It interacts with cells and initiates the natural physiological processes involved in the healing and regeneration of tissue. It supports the delivery and the retention of biochemical factors facilitating proper cell attachment and migration [148]. Biomaterial types and processing techniques are important for the properties of the resultant scaffolds. Moreover, a scaffold necessitates being either bioresorbable or pro-regenerative, thus displaying attributes such as notable porosity, a substantial surface area-to-volume ratio, a distinct geometry, and a pliable nature to adapt to the wound’s contour. These scaffolds can be fashioned from natural, synthetic, or hybrid polymers, and are amenable to functionalization with various agents aimed at augmenting cellular reactions and accelerating the wound healing trajectory. The manufacturing of these scaffolds can be achieved through techniques like electrospinning or 3D printing [149].

### 3.7. Hydrocolloids

Among advanced dressings, those based on hydrocolloids create a moist environment and absorb medium amounts of exudate. They are available in plaques and pastes and promote the growth of granulation tissue, favoring the healing process. In the presence of exudate, they absorb the malodorous liquid and produce a gel, which is why they are so used in the treatment of pressure sores. A hydrocolloid dressing consists of a thin dressing that contains gelling agents in an adhesive compound, laminated to a flexible, water-resistant outer layer. The sheets are self-adhering and available with or without an adhesive border, in different thicknesses and shapes pre-cut for various areas of the body such as the sacrum, elbows, and heels. Hydrocolloid dressings are occlusive, thus providing a moist healing environment, autolytic debridement, and isolation, without permeability to bacteria and other contaminants. They are easy to apply, self-adherent moldable dressings (do not adhere to the wound, only to the intact skin around the wound) that can be used under venous compression products. Essential to keep the decubitus wound clean, they can remain in place for 3 to 7 days (depending on the amount of wound exudate), greatly limiting the trauma produced by the operation of changing the dressing while avoiding disturbing the healing [150]. The ideal wounds for the use of hydrocolloid dressings are low-exudating and non-infected skin lesions. They can also be used as an alternative tool in wound prevention, to protect fragile skin or recently healed wounds with re-epithelialized skin. Among the best hydrocolloid dressings, there is DUODERM CGF; it retains exudate very well and it is used in the treatment of wounds with low exudate production [151].

### 3.8. Foam Dressings

Foam dressings are crafted from polymer solutions, creating structures with open cells capable of retaining fluids. These foams are commonly composed of materials like PU or silicone. PU foam dressings typically consist of two or three layers, featuring a hydrophilic surface in direct contact with the wound and a highly absorbent hydrophobic layer. These dressings offer multiple benefits such as thermal insulation for the wound bed, facilitating the exchange of water vapor and oxygen, effective dispersion of exudate within the absorbent layer, and preventing its escape to the external environment. In some instances, PU foam dressings are available in the form of cavity dressing-chips enclosed within a perforated polymeric film membrane.

On the other hand, silicone foams are produced using a silicone elastomer derived from the combination of two liquid components. When mixed, these components create a foam that conforms to the contours of the wound, resulting in a soft, open-cell foam dressing. These silicone foam dressings also contribute to efficient fluid management and wound protection [152]. In conclusion, the major advantages of foams are that they promote permeability to vapor and gases and create an impervious barrier to fluids and bacteria, protecting the wound. The main forms of dressing materials are shown in Table 1. 

## 4. Bioactive Molecules

**Figure 7:**
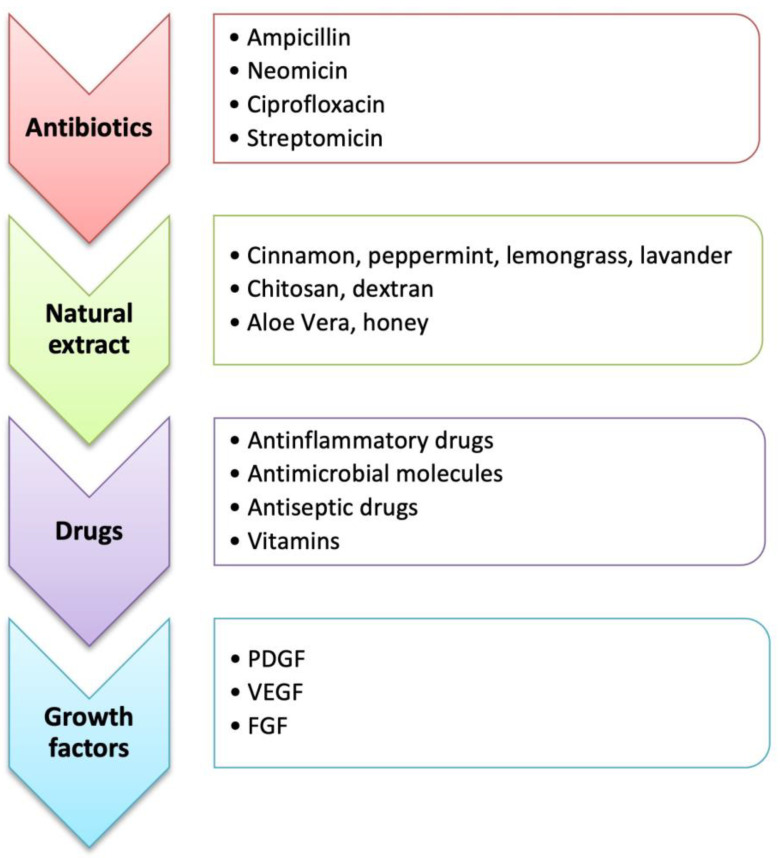
Main bioactive molecules used in wound healing.

The incorporation of biomolecules or active pharmaceutical ingredients (APIs) in different types of wound dressings and structures prevents skin infections and promotes the healing process. The 3D wound dressing architecture, which mimics the ECM and has a porous structure, can be functionalized with bioactive molecules. Different bioactive molecules can be included in a polymeric matrix, highlighting the antibacterial biomolecules (e.g., antibiotics, silver NPs, and natural extracts-derivate products) and molecules capable of enhancing the healing process (e.g., growth factors, vitamins, and anti-inflammatory molecules) (Figure 7) [2,157].

### 4.1. Antibiotics 

In clinical therapy, antibiotics are usually orally administered to prevent microbial colonization/contamination of open wounds. To contrast many antibiotics’ drawbacks, such as their rapid elimination from the blood stream or the narrow therapeutic index, their topical administration has been explored by loading them in several polymeric matrices [158,159]. Different antibiotics have been incorporated in polymeric matrixes, such as ciprofloxacin [54], gentamycin [160], tetracycline [161], and silver sulfadiazine [162].

For example, Alavarse et al. developed tetracycline hydrochloride (TCH)-loaded PVA/Chitosan (80/20 *v*/*v*) electrospun nanofibers. The polymeric nanofibers were produced by the electrospinning method, and smaller diameters were recorded after the addition of chitosan and TCH. Furthermore, the TCH-loaded nanofibers showed a uniform distribution of the drug along the nanofibers and beads. They maintained the inhibition effect on Gram-negative *E. coli*, as well as Gram-positive *S. epidermidis* and *S. aureus*, sustained by a burst drug liberation within the first 2 h, highlighting their potential application in the treatment of different kinds of wounds [163].

### 4.2. Silver Nanoparticles

Recent advances in nanotechnology-based therapies have focused on fighting microorganisms’ multidrug resistance. In particular, AgNPs are emerging as a promising alternative to the use of antibiotics due to their biological and chemical properties. They have excellent antimicrobial properties as tested against many different strains of bacteria [164]. Their mechanism of action consists of the adhesion to the bacterial cell wall and the subsequent disruption of the cell membrane. Moreover, silver ions are able to produce ROS, inducing oxidative stress [165]. Ruffo et al. loaded AgNPs into a CMC-based hydrogel for chronic wound treatment. NPs were released in a slow and sustained manner with a wound closure percentage of 75 ± 0.3% due to the modulation of cytokine production and the mitigation of the inflammatory process by AgNPs [166]. The observed antibacterial activity makes AgNPs a good candidate for the development of wound dressings, some of which are already commercially available: Acticoat^®^ [167], Aquacel Ag^®^ [168], Silvasorb^®^ [169], and SilvercelTM [170].

### 4.3. Natural-Extract-Derived Products

Plant-derived extracts and essential oils (EOs) have undergone evaluation as promising reservoirs of innovative antimicrobial agents [171]. The antimicrobial attributes of oil-based extracts primarily stem from the existence of bioactive elements like terpenes, terpenoids, and additional aromatic and aliphatic components. The hydrophobic nature of these constituents facilitates the separation of lipids within the cellular membrane, thereby heightening membrane permeability. This process ultimately culminates in the demise of bacterial cells, as essential molecules and ions leak out. [172]. Therefore, essential oils are able to overcome bacterial resistance to antibiotics or multi-drug resistance. Liakos et al. reported the incorporation of three different (cinnamon (CN), lemongrass (LG), and peppermint (PM)) EOs into cellulose acetate (CA) electrospun nanofibers [173]. The most-extracted compounds used for biomedical applications include lavender, tea tree, propolis, or aloe vera. They showed antimicrobial and antioxidant activities for many bacteria [174]. Propolis extract, which is a resinous blend produced by bees, also exhibits properties that counteract bacteria, fungi, viruses, oxidation, and inflammation. These effects stem from a diverse array of chemical constituents present, including aglycone flavonoids, phenolic acids, and ester derivatives [175,176]. Aloe vera and chitosan are additional instances of natural sources featuring antimicrobial attributes due to their chemical makeup. Amino acids, salicylic acid, ascorbic acid, vitamin A, and vitamin E, present within aloe vera, contribute to the extract’s ability to combat bacteria, curb inflammation, and offer antioxidant benefits [177]. 

### 4.4. Drugs 

The initial stage of the wound healing process, known as the inflammatory phase, commences with hemostasis, aiming to prevent blood and fluid loss while also eliminating deceased tissues and guarding against infection. Within this phase, essential roles are fulfilled by inflammatory cells—namely, monocytes, macrophages, and neutrophils. They are pivotal in purging the wound site, responsible for disposing of non-viable cells, neutrophils laden with bacteria, damaged extracellular matrix (ECM), and bacteria themselves. However, in cases where the inflammatory process persists over a protracted period, the sustained attraction of neutrophils and macrophages results in excessive production of inflammatory mediators, free radicals, and cytotoxic enzymes. This disrupts natural healing mechanisms and inflicts harm upon the neighboring skin area. To address this issue, the incorporation of anti-inflammatory agents into wound dressings emerges as a pivotal strategy for treating skin injuries. These agents hold the potential to hinder the development of persistent inflammation and counteract the accumulation of free radicals.

Among the most frequently employed substances for alleviating pain or addressing chronic wounds are anti-inflammatory compounds like ibuprofen [178], ketoprofen [179], and diclofenac [180]. Additionally, corticosteroids such as dexamethasone are utilized in this context. [181].

In the last few years, most of the attention has been focused on curcumin. It is able to reduce the release of inflammatory cytokines from monocytes and macrophages (interleukin IL-8 and tumor necrosis factor-α TNF-α) and it inhibits the enzymes associated with inflammation, such as cyclo-oxygenase 2 (COX-2) and lipoxygenase (LOX) [156].

### 4.5. Growth Factors

Growth Factors (GFs) are dynamic polypeptides with the capacity to oversee cellular growth, differentiation, proliferation, migration, and metabolism throughout the progression of wound healing. The entire spectrum of wound healing stages is under the sway of a diverse array of GFs and cytokines, prominently encompassing epidermal growth factor (EGF), platelet-derived growth factor (PDGF), transforming growth factor-β (TGF-β), fibroblast growth factor (FGF), and vascular endothelial growth factor (VEGF) [182]. These molecules assume a pivotal role in the creation of granulation tissue, fine-tuning the inflammatory response (as exemplified by PDGF, TGF-β), instigating the release of interleukins (such as IL-1 and IL-6), and fostering angiogenesis (e.g., EGF and VEGF). However, the topical application of GFs encounters certain limitations, including diminished in vivo stability, constrained permeation through the skin barrier, premature removal via exudation before reaching the wound site, and potential undesired effects due to elevated localized and/or systemic concentrations. Consequently, the encapsulation of GFs within nanofibers or alternative structures is increasingly seen as an alluring strategy to enhance the wound healing process [155].

## 5. Methods of Preparation for Wound Dressings

Wound dressings are produced by different methods which depend on the desired structure and the materials used. The three methods commonly employed are solvent casting, electrospinning, and extrusion-type three-dimensional (3D) printing. 

### 5.1. Solvent Casting Technique

**Figure 8:**
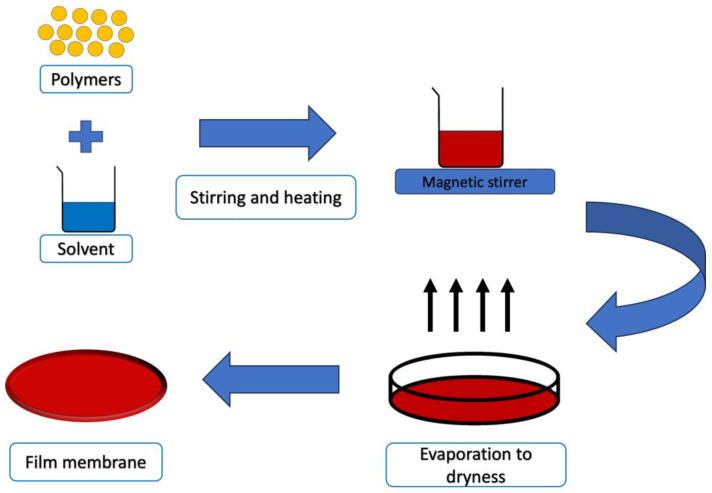
Solvent casting technique.

The solvent casting technique involves creating polymeric films by drying viscous solutions containing polymers, additives, and active substances in a uniformly spread layer (Figure 8). While this method is straightforward, it can be time-intensive, and the properties and stability of the resulting films are primarily influenced by the chosen materials [154]. Typically, polymers are dissolved or dispersed in an organic solvent, which is then poured onto a supporting surface. As the solvent evaporates during drying, a solid layer forms on the substrate. Occasionally, particles of specific sizes, often salts, are incorporated into the solution. The dried mixture is then shaped into its final form. Alternatively, the composite material can be immersed in a bath to dissolve the particles, leaving behind a porous structure. These solvent-cast polymer films exhibit enhanced structural integrity and are well-suited as the foundational layer in multi-layered formulations [183].

In a recent study, a PVA/dextran hydrogel was developed using the solvent casting method in order to provide an efficient wound dressing. Zataria essential oil was loaded in the hydrogel as an antioxidant and antibacterial agent. PVA/dextran gels were crosslinked with GA and the mixture was poured in a petri dish and stored at 60 °C for 5 h. In vitro studies revealed the dressing’s ability to prevent wound inflammation and infection at the same time [184].

### 5.2. Electrospinning

Electrospinning is a versatile technology utilized to create polymeric fibers with excellent diffusion properties and a high surface area to volume ratio. These attributes make it valuable for wound care applications, aiding in controlling bleeding, absorbing excess wound fluid, and fostering tissue regeneration [185]. 

**Figure 9:**
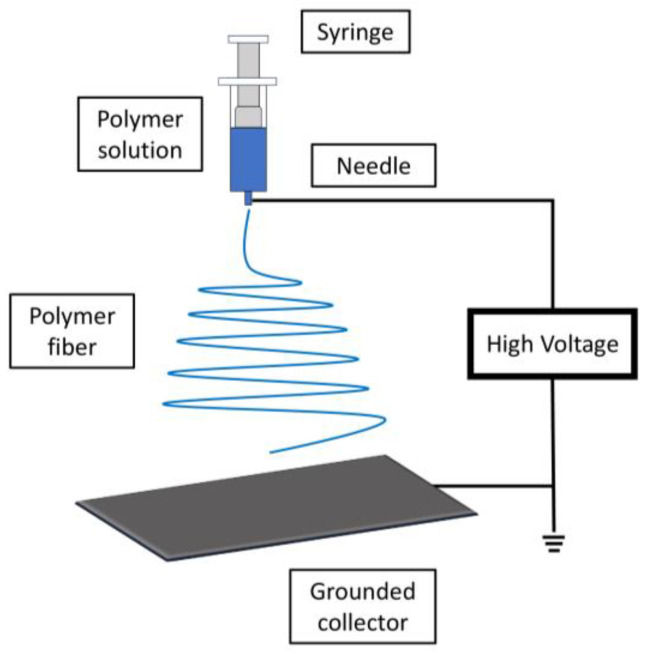
Electrospinning system.

The electrospinning procedure employs a high Direct Current (DC) voltage, typically within the range of several kilovolts (10–20 kV). This voltage is applied to generate electrical charges within a stream of polymer solution, which then dries, resulting in the formation of polymer nano fibers (Figure 9).

The stability, consistency, and production efficiency of the electrospinning process hinge upon the compatibility of various components: the chosen polymer(s), solvent(s), and any additional additives or active substances.

Indeed, the process of electrospinning involves several key steps to create polymer nanofibers for wound care applications: the chosen polymer is first dissolved in a suitable solvent to form a homogeneous solution. The properties of the polymer, solvent, and their compatibility play a significant role in achieving successful electrospinning. The polymer solution is then carefully introduced into the syringe tube that is part of the electrospinning setup. The solution will be dispensed through a small-diameter needle or spinneret. The positive terminal of a DC power supply is connected to the hollow needle or spinneret, while the negative terminal is connected to a metal collector, often in the form of a rotating drum or plate. The solution within the syringe is subjected to a high electric field generated by the voltage difference between the needle and the collector. The electric field causes charges to accumulate at the surface of the droplet of polymer solution at the tip of the needle. The electric field forces the charged droplet to overcome the surface tension and elongate into a conical shape known as a Taylor cone. A fine jet of polymer solution is emitted from the tip of the cone. As the jet travels toward the collector, the solvent evaporates, leaving behind a solid fiber. During its flight from the needle to the collector, the solvent in the jet evaporates due to the temperature and pressure conditions in the surrounding environment. This process leads to the solidification of the polymer into a continuous nanofiber. The nanofibers are collected on the metal collector, which may be a rotating drum or stationary plate. The fibers form a nonwoven mat on the collector’s surface. Stable environmental conditions, including temperature and humidity, are crucial to ensure the quality and uniformity of the nanofibers. Factors such as the distance between the needle and collector, solution vapor pressure, and chamber temperature are optimized to achieve the desired fiber properties.

Overall, electrospinning is a complex yet highly controlled process that enables the production of fine polymer nanofibers with tailored properties for various applications, including wound care. The resulting nanofibrous mats can provide enhanced capabilities for wound healing, such as improved absorption, moisture management, and cell interaction [186].

Habibi et al. reported the synthesis of a bi-layer chitosan/PVA nanofiber loaded with bupivacaine and mupirocin. Smooth and uniform nanofibrous mats were obtained using an electrospinning apparatus. Bupivacaine was used as a topical anesthetic to reduce the pain, while mupirocin exerted antibacterial activity. Cell viability studies highlighted the ability of the engineered wound dressing to mimic the ECM and histopathology studies confirmed its potential to accelerate the wound healing process [187]. 

### 5.3. Melt-Blowing

Melt blowing is a versatile technique used in the preparation of wound dressings. In this process, thermoplastic polymers are melted and then forced through fine nozzles to form microfibers. These microfibers are rapidly cooled by high-velocity air streams, causing them to solidify and create a nonwoven web of interconnected fibers. The resulting nonwoven material has a unique combination of porosity, surface area, and mechanical strength, making it suitable for wound dressing applications [188]. Melt blowing offers several advantages for wound dressing production. The technique allows for the incorporation of various materials, such as antimicrobial agents or growth factors, into the polymer melt, enhancing the functional properties of the dressings. The resulting nonwoven structure promotes moisture management and breathability, creating a favorable environment for wound healing [189]. Additionally, the scalability and efficiency of the melt blowing process make it suitable for large-scale production of wound dressings with consistent quality [190].

The versatility of melt-blown wound dressings makes them suitable for various wound types, from minor abrasions to more severe injuries. The ability to tailor the material properties and composition to specific wound requirements highlights melt blowing as a valuable method for preparing wound dressings that support optimal healing conditions [191]. 

Wang et al. developed microfiber-based nonwovens using a melt blowing technique, combining PLA and PEG with sodium dodecyl sulfate (SDS). The influence of melt blowing technology parameters, including die temperature and hot air pressure, was examined in relation to the structure and characteristics of the microfibers nonwovens. Elevating either the die temperature or the hot air pressure led to improved melt flow, facilitating thorough fiber stretching and gradually enhancing the fabric’s mechanical properties. Decreased fiber diameters yielded smoother surfaces and smaller pores, resulting in increased liquid pressure generation, thereby enhancing fabric wettability. The findings demonstrated the potential of utilizing PLA/PEG/SDS microfiber-based nonwovens as raw materials for wound dressing production, owing to their remarkable liquid absorption capacity. Furthermore, their inherent ability to undergo natural degradation post-use offers an environmentally friendly solution, reducing their environmental impact [192].

### 5.4. Thermal Annealing

Thermal annealing is a process used in wound dressing preparation to enhance the mechanical properties, stability, and functionality of the materials used. It involves subjecting a polymer film or material to controlled heating and cooling cycles to induce structural changes at the molecular level instead of using chemical and harmful cross-linkers. This process can lead to improvements in the wound dressing’s properties, making it more suitable for its intended application [193]. 

In a recent study, electrospun PVP-based fibers loaded with hydroxycinnamic acid derivatives (p-coumaric and ferulic acids) were synthesized. The fibrous mats were transformed into hydrogels via thermal annealing. The phenolic compounds in the fibers maintained their antioxidant activity even after annealing and the fibers demonstrated antioxidant effects in vitro and cell experiments, protecting against oxidative stress. The fiber mats showed suitable swelling properties, forming hydrogels when immersed in water. Moreover, in mice with UV-B-induced burns, the fibers reduced pro-inflammatory marker levels. These thermally treated PVP fibers loaded with phenolic acids showed potential as active skin dressing materials for healing skin injuries with oxidative stress [126]. 

### 5.5. 3D Printing Technology

The technology of 3D printing creates layers of materials to form a three-dimensional structure. This technique allows you to print different materials, from plastic to metal, from resins to different polymers. The use of 3D printing for wound dressing made possible the generation of skin tissue and constructs similar to physiological skin. It has been used for on-demand therapies for the production of complex pharmaceutical forms. Today, 3D printing technology is the most interesting manufacturing technology thanks to the possibility of obtaining highly customizable products. For this characteristic, a 3D printer is able to produce a product with a bottom-up fabrication by a layer-by-layer method. The object to be printed is created using a computer-aided design (CAD) software package which is then exported as a file to be printed. The exported file splits the 3D object into a series of layers and then starts printing. The growth in the number of publications over the last years confirms the interest in 3D printing. It can be used in many areas, from engineering to the biomedical and pharmacological fields. In particular, in the development of pharmaceutical and biomedical products, 3D printing can be used for dressing up three-dimensional supports covered in or containing active ingredients to be delivered to the skin [194]. The emergence of 3D printing technology and its diverse applications in various fields offers a multitude of benefits, particularly in enhancing the quality of life for patients, promoting treatment adherence, and boosting the effectiveness of therapies, notably in cases of chronic wounds [195]. The intrinsic qualities can closely mimic the natural environment of the skin, thereby fostering an optimal milieu for the treatment of diseases and injuries [196].

Recent advancements have seen 3D printing employed for the production of scaffolds and patches tailored for transdermal applications [194]. Furthermore, as bioprinting techniques have continue to advance, enabling the printing of living cells, these systems have been harnessed for the realm of skin tissue engineering [197]. 

The basic components of a 3D system can be divided in three groups: hardware (which is the 3D printer itself), software (used to communicate with hardware and also allow conversation of CAD images into stereolithography images which are recognized by the printers), and materials used to print objects [198].

**Figure 10:**
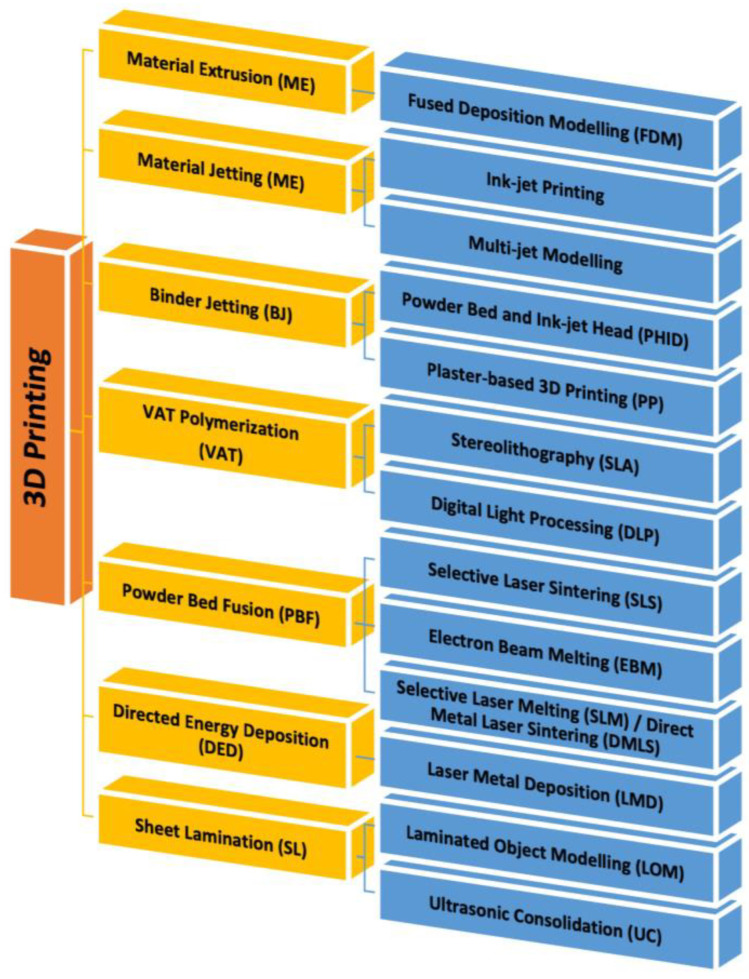
Classification of different 3D Printing processes.

Based on the specific application and the desired material to be used, three main groups of 3D printing techniques can be distinguished: extrusion-based methods, powder-based methods, and photopolymerization methods. Each of these groups include different approaches with slight mechanical or chemical variations, such as Material Extrusion (ME), Binder Jetting (BJ), Powder Bed Fusion (PBF), Sheet Lamination (SL), Material Jetting [136], VAT polymerization, or Directed Energy Deposition (DED) (Figure 10) [199,200].

ME is an additive manufacturing methodology where a spool of material (usually thermoplastic polymer) is pushed through a heated nozzle in a continuous stream and selectively deposited layer by layer to build a 3D object. In fact, it is also known as fused filament fabrication or fused deposition modeling [201]. BJ is the second most used 3D printing technology and is a process in which a binder is printed onto a powdered material, binding it together to develop a 3D printed structure [202]. PBF uses an electron beam, laser, or other heat source to selectively consolidate the powder in each layer into 3D objects. PBF technologies comprise Selective Laser Sintering (SLS), Selective Laser Melting (SLM)/Direct Metal Laser Sintering (DMLS), Electron Beam Melting (EBM), and Multi-Jet Fusion (MJF) [203]. SL is another process in additive manufacturing in which a laser is used to cut the material, joined to a substrate with a heated roller, to the desired shape [204]. MJ is a process in which tiny droplets are propelled from specialized drop-on-demand print heads. These print heads employ thermal, piezoelectric, or electromagnetic mechanisms to create pressure pulses. The resulting droplets are meticulously positioned on a substrate at designated spots. Following each layer’s deposition, UV light triggers polymerization, solidifying the material. This layer-by-layer approach gradually constructs a three-dimensional object, with successive layers fusing together to form the final structure [205]. Vat polymerization is a method used to print 3D objects by using photopolymerization. The process exposes liquid polymers to UV light to turn liquid into solids. Stereolithography [103] is the primary technique of vat polymerization, and it enables fast production with a precise architecture [206]. Finally, DED uses a focused energy source, such as a laser or an electron beam, to fuse the feedstock material and continuously deposit it, layer by layer [207].

#### 3D Bioprinting

3D bioprinting technology is widely employed in regenerative medicine. It is used to generate different types of tissue, creating grafts directly on the patient’s skin in the shortest possible time and at reduced costs. Bioprinting creates customized treatments and minimizes the risk of organ rejection after transplantation. It is a type of additive manufacturing that integrates living cells into the printing procedure, often in conjunction with biomaterials. This field can be categorized into two key technologies: extrusion and inkjet bioprinting [208]. In extrusion bioprinting, a continuous flow of bioink—a viscous solution containing living cells and biomaterials—is precisely dispensed in strands. These strands are layered on top of one another to create intricate three-dimensional structures [209]. In inkjet bioprinting, droplets containing cells are deposited with or without the addition of biomaterials onto a receiving substrate [210]. Natural and synthetic polymers, metal, and ceramic are the most common materials used for this application [211]. 

In the last few years, 3D bioprinting has played a key role in tissue regeneration. It allows the development of biocompatible structures able to mimic the natural systems with good reproducibility in terms of size, shape, geometry, and orientation with high precision, while additionally providing the option to use a variety of bioink material and cell types (cells, growth factors, biomolecules, and biopolymers) in a controlled manner. A bioink should be biocompatible to enhance cell growth and proliferation and possess high chemical and physical fidelity to preserve its shape after deposition and post crosslinking [212]. Many natural polymers, such as collagen, chitosan, alginate, and cellulose, as well as several synthetic biopolymers have been used as bioinks to develop constructs with a specific design [213]. Finding application in wound treatment, 3D printing enables the synthesis of 3D devices able to enhance skin regeneration and the healing process. Recently, Hao et al. developed a gelatin–alginate-based hydrogel using 3D extrusion bioprinting, and hUC-MSCs (human umbilical cord mesenchymal stem cells) were laden in the sterilized bioink. The hydrogel showed good biocompatibility properties, ensuring cell functions in therapeutic applications. Moreover, a full-thickness skin defect repair experiment in mice was performed and the 3D printed cell-laden hydrogel significantly promoted wound healing by modulating inflammatory response and accelerating angiogenesis [214]. A double-crosslinked alginate/chondroitin sulfate patch was realized by the 3D printing approach, adding acrylated VEGF to the bioink. VEGF represents an alternative for skin wound treatment because of its ability to regulate angiogenesis during organism trauma repair. The hydrogel patches were first physical crosslinked using CaCl_2_ and then exposed to UV light for photocrosslinking. The VEGF-loaded patch prolonged the growth factor release, exhibiting excellent angiogenic ability and promoting wound healing [215].

## 6. Conclusions and Future Directions

The skin is a complex organ with a well-defined structure consisting of multiple layers and different cell types. In the latest few years, the field of wound healing has been extremely active. Several wound dressing materials have been developed to promote the healing process and protect the wound from infection. Traditional wound dressings often proved to be ineffective against bacterial contamination, even though they are still used for their low cost and ease-of-use. Currently, different types of advanced wound dressings are available to provide a moist environment to the wound and act as a barrier against bacteria penetration. They are usually made of natural or synthetic polymers, known for their biocompatibility, biodegradability, and non-toxicity. Bioactive molecules, such as antibiotics, growth factors or natural extract, are normally added to the formulations to accelerate the healing process, especially in chronic wounds. 

Wound dressing materials can be manufactured using various methods, like solvent casting, electrospinning, and 3D printing. The final product will differ in size, structure, thickness, and mechanical properties according to the preparation method. In particular, 3D bioprinting stands as a groundbreaking strategy aimed at fabricating biomimetic tissues infused with bioactive compounds, diverse biomaterials, and vital living cells. These engineered constructs boast mechanical resilience akin to natural tissue, while also catering to the metabolic and physiological demands of living systems, underscoring their remarkable reproducibility. However, it is important to note that each of these methodologies carries distinct advantages and limitations, manifesting through discrepancies in aspects such as resolution, strength, mechanical attributes, and the accuracy of the printed structures.

In this review, we provided an overview on the synthesis and classification of wound dressing materials. They have been classified according to their sources (natural or synthetic) and physical form. A variety of bioactive molecules can be added to modern dressings to enhance their healing activity. The main synthesis methods for wound dressings have been presented to highlight their benefits in the treatment of injured skin tissue. 

The future trends in wound dressing development should focus on the use of more cost-effective and “green” approaches, as well as the improvement of the effectiveness of the component materials. The synthesis of new hybrid polymers and more efficient cross-linking methods should be taken into consideration in order to expand the efficacies of wound dressings. Nevertheless, the flexibility, speed, and ability to create complex and different constructs has recently made 3D printing a potential alternative in wound care. Allowing for the development of dressings with customized parameters (size, dose, swelling properties, drug release profile), 3D printing is able to increase patient compliance and, at the same time, improve the effectiveness of the therapy. The use of this technique in wound healing provides many advantages and makes possible the avoidance of several drawbacks of traditional dressings, such as skin irritation, high cost of production, discomfort, or difficulty of application.

## Figures and Tables

**Table 1:** Classification of wound dressings according to their physical form.

Dressings Material Form	Materials/Polymers	Bioactives	Results	Limitations	References
Bandages	Cotton wool; gauze cotton with polymers as PCL	Antibiotics and anti-inflammatory agents	Primary and secondary dressings	Discomfort during dressing changes, poor adhesive properties, and low drainage level for the wound	[37]
Lyophilized wafers	Synthetic polymers: PU	Antibiotics and growth factors	Adhesive dressings	Quite fragile, difficulty in application, lack of flexibility	[142]
Hydrogels	Natural and synthetic polymers capable of creating tridimensional network: PEG, chitosan, PVA, PVP	Antimicrobial agents and several actives	Secondary dressings in the form of insoluble aqueous gels facilitated autolytic debridement, protecting against desiccation and creating an environment conducive to effective wound healing	Limited absorption capacity, need to be reapplied relatively frequently, low mechanical strength	[78,79,80,87,92,96,98,109,121,127,153]
Films	A translucent, typically PU, thin film	Antimicrobial agents	Moist environment. Waterproof and transparent Semi-permeable to oxygen and water vapor while effectively blocking liquids and bacterial contamination, eliminating the need for an additional dressing	Insufficient mechanical strength, removal can be painful, limited absorption of odor and exudate	[56,91,145,154]
Patches	Combination of biomaterials: chitosan, cellulose, HA, PVA, PCL	Antiseptics such as AgNPs and other agents as polyhexamethylene biguanide, gold NPs, bacitracin, and metronidazole. Antioxidant agents isolated from plants such as Bletilla striata, Calendula officinalis	Possessing therapeutic attributes such as adhesion, absorption, mechanical support, and robustness within a multi-layered structure	Can be occlusive thus trapping moisture against the wound, limited exudate management capabilities, allergic reactions to the adhesive used in patches	[146]
Scaffolds	Natural and synthetic polymers such as chitosan, PVA, and surfactants.	Active drugs such as anti-inflammatories, antibiotics, and antiseptics.	Primary and secondary dressings designed to replicate the characteristics of the skin, effectively acting as skin mimics or pseudo-skin	Difficulty in application, can provide surfaces where bacteria can colonize, increasing the risk of infection	[62,67,85,102,155]
Hydrocolloids	Gel with agents such as elastomers, Gelatine, pectin, and adhesive elements	Local anesthetics as lidocaine	Occlusive dressings. Used in dried, black eschars and for grade 1 and 2 pressure ulcers and moisture lesions	Do not absorb blood or bacterial infection, do not promote autolytic debridement, difficulty in removal	[150]
Foam dressing	Hydrophilic PU	Antimicrobials and other actives to form a barrier from microorganisms	Utilized for wounds with moderate to high drainage levels, these dressings are exceptionally absorbent, offering cushioning, protection, and conformability to various body contours. Their absorbent nature reduces the need for frequent replacement, while their easy removal adds to their convenience	Limited adhesion to dry wound surfaces, might saturate quickly if the wound has very high exudate levels, skin irritation or allergic reactions to the adhesive or foam material used in these dressings	[134,135]
Sponges	Hydrophilic natural and synthetic polymers such as cellulose, collagen, sodium alginate, PVA or PEG	Antimicrobial or hemostatic bioactive molecules	Used to absorb exudate or blood and to clean the wound	Poor mechanical property, variable absorption capacity, “macrosponge” formation, do not maintain a controlled-moisture environment	[57]
Membranes	Chitosan, alginate, PVA	Antimicrobial or growth factors encapsulated	Used for non-adherent wound, secondary damage, and injury without scarless wound healing	Limited adsorption, might not conform well to wounds with deep crevices or irregular shapes, might not make full contact with the wound bed	[73,90]
Fibers	Cellulose	Antibacterial agents	Tissues engineered	Poor mechanical properties, easy to saturate thus requiring frequent dressing changes, variable absorption capacity	[69,70,128,156]
